# Allelopathic Effect of the Invasive *Ludwigia hexapetala* on Growth of Three Macrophyte Species

**DOI:** 10.3389/fpls.2018.01835

**Published:** 2018-12-18

**Authors:** Gabrielle Thiébaut, Lise Thouvenot, Hector Rodríguez-Pérez

**Affiliations:** ^1^ECOBIO, UMR 6553 CNRS, Université de Rennes 1, Rennes, France; ^2^German Centre for Integrative Biodiversity Research (iDiv) Halle-Jena-Leipzig, Leipzig, Germany; ^3^Institute of Biology, Leipzig University, Leipzig, Germany

**Keywords:** allelopathy, macrophytes, native, exotic species, functional traits

## Abstract

The release of allelochemicals by plants can affect the performance of other organisms positively or negatively. We tested the effects of aqueous extracts and leachates derived from the leaves and roots of the invasive water primrose (*Ludwigia hexapetala*) on one submerged native species – *Ceratophyllum demersum*, and two exotic species – the submerged *Egeria densa* and the emergent growth form of *Myriophyllum aquaticum*. The effect of the aqueous extracts and leachates of *L. hexapetala* on photosynthetic yield, growth (i.e., relative growth rate, leaf area), root length, and length of the lateral shoots of each species were analyzed in spring and in autumn. In autumn, an allelopathic effect was established on the traits of the three macrophytes species. The root extracts stimulated leaf area and the photosynthetic yield of *C. demersum* and of *E. densa*, whereas leaf treatments (leachates and extracts) and root leachate reduced the leaf area of *M. aquaticum*. The autumnal root leachate of *L. hexapetala* decreased the relative growth rate of *C. demersum*, whereas it had no effect on the two others plants. The root extract increased the length of lateral branches of *M. aquaticum* in autumn, suggesting a positive effect of *L. hexapetala* on the lateral growth of *M. aquaticum.* Three main allelochemicals were identified in leaves: quercitrin, prunin, myricitrin. The concentrations of these allelochemicals were greater in the leaf extract taken from *L. hexapetala* in autumn than in spring, and those found in the leaf leachates for both seasons. This assessment of autumnal allelopathy could help to explain the patterns of plant community succession in invaded areas.

## Introduction

The allelochemicals released by organisms into the environment, also called “allelopathy” (Rice, 1984; Elakovich and Wooten, 1989) have beneficial or detrimental effects on neighboring organisms (e.g., phytotoxicity, soil sickness). Allelochemicals are released directly from plants through different mechanisms, such as root exudation, leaching of aerial parts, and volatilization, and also passively through plant decomposition. The role of allelopathy on the structure and composition of biological communities is relatively unexplored in freshwaters (Kulshretha and Gopal, 1983; Agami and Waisel, 1985; Elakovich and Wooten, 1989; Gross, 2003; Dandelot et al., 2008). Nevertheless, some studies showed that several aquatic macrophytes (i.e., *Elodea nuttallii*, *Myriophyllum spicatum*) can impact the phytoplankton (Gross, 2003) and inhibit germination and/or seedling growth (Gopal and Goel, 1993; Dandelot et al., 2008) *via* the release of allelochemicals. Bioassays, using plant extracts (i.e., leachates, exudates), are one of the most common methods used to assess the allelopathic effects of plants. Overall, they have generally only tested the allelopathic potential of plants at one point in time, even though the synthesis of allelochemicals and their concentrations in the plant fluctuate throughout the year (Helmig et al., 2013; Santonja et al., 2018). Indeed, the seasonal variation in allelochemicals could be explained by the fluctuations of abiotic and biotic parameters, i.e., climatic conditions (Petrussa et al., 2013), the presence of herbivores and/or pathogens (Gatti et al., 2014; Silva et al., 2014) and stage in the life-history of the plant (Lombardo et al., 2013; Santonja et al., 2018). The seasonal dependence of plant allelopathic interactions is still understudied, although it could help to explain exotic plant establishment, their spread and plant community succession in invaded areas. Indeed, the potential allelopathy of exotic plants could favor their establishment and their spread into their introduced range (Callaway and Ridenour, 2004). Many exotic plants could synthesize unknown allelochemicals and release them into the native community (c.f. “Novel Weapons Hypothesis,” Callaway and Ridenour, 2004). These novel allelochemicals could inhibit the growth of native plants and thus improve the growth of the invasive species (Callaway and Ridenour, 2004; Kim and Lee, 2011). In this way, as stipulated in the Invasional Meltdown Hypothesis (Simberloff and Von Holle, 1999), the introduction of one species may favor the introduction and spread of one or more other exotic species. However, they can also affect other invasive species negatively, if they do not come from the same biogeographical area.

This paper is focused on the potential allelopathic effect of the invasive water primrose *Ludwigia hexapetala* (Hook. and Arn.) Zardini, H. Y. Gu and P. H. Raven. (syn. *L. grandiflora* subsp. *hexapetala*) on three macrophytes species. The amphibious *L. hexapetala*, native to South America, was introduced to Southern France in 1830 (Thouvenot et al., 2013a). Once established, it formed dense mats in freshwaters, on riverbanks and in meadows (Thouvenot et al., 2013a). Its invasive success could be partially explained by the release of secondary metabolites into the recipient community (Dandelot et al., 2008; Santonja et al., 2018) which could limit the growth of native plants. Indeed, the presence of *L. hexapetala* reduces both the plant richness and the abundance of the native species such as the submerged *Ceratophyllum demersum* or some emergent species (*Alisma plantago-aquatica*, *Lycopus europaeus*; Stiers et al., 2011). The water primrose *L. hexapetala* exhibits a horizontal growth stage over water with small round leaves and a growth stage with erect elongated leaves. We used root and leaf leachates and aqueous extracts of *L. hexapetala* from individuals collected in spring and in autumn to analyze the impact of these solutions on the traits of one native species [*C. demersum* L. (Ceratophyllaceae)], and on two exotic species [*Myriophyllum aquaticum* (Vell.) Verdc. (Haloragaceae)], and [*Egeria densa* Planch. (Hydrocharitaceae)]. Our aim was to gain a better understanding of the responses of other macrophytes species to the invasive species. We hypothesized (1) that leaf and root treatments would induce a decrease in the growth of the three target species and (2) that the effects of *L. hexapetala* on the target plants would change according to the season.

## Materials and Methods

### Plant Materials

The amphibious Parrot’s Feather, *M. aquaticum*, native to South America was introduced as ornamental plant into France in approximately 1880 (Sheppard et al., 2006). The species can cause severe problems in Southern Europe (Les and Merhoff, 1999), in the southern states of the United States, in South America (Fernandez et al., 1993), in New-Zealand and Australia. Once introduced into a new region it spreads rapidly, primarily by vegetative stem fragmentation. It is often found in eutrophic water bodies (small streams, ponds, slow-running waters and irrigation channels). Stems of *M. aquaticum* float out over the water surface to form dense mats from which emergent shoots arise (Hussner, 2009). This species has demonstrated a potential inhibitory effect on neighboring plants (Elakovich and Wooten, 1989).

The Brazilian water-weed, *E. densa*, is a native, submerged macrophyte coming from Argentina, Brazil, and Uruguay. Historically, this species was introduced outside its native range by the aquarium trade. It has been in cultivation in France at least since 1919. It was observed in the field in France in 1960 (Cook and Urmi-König, 1984) and is considered as a nuisance in Central and North America, in Europe and in Australasia (Cook and Urmi-König, 1984). *E. densa* reproduces vegetatively from plant fragments. It has a massive build-up of biomass, allowing it to become highly invasive. Its dense mats reduce recreational activities and crowd out native species, as well as altering the hydrology. Several authors (Nakai et al., 1999; Vanderstukken et al., 2011; Espinosa-Rodríguez et al., 2016) have found allelochemicals which affect phytoplankton negatively.

The European Coontail (*C. demersum*) is a rootless submerged plant found in freshwaters with moderate to high nutrient levels. This plant drifts in the water without being attached to the sediment and the species is usually well equipped to capture high to very high nutrient levels from the surrounding water (Denny, 1972). According to several authors (Kleiven and Szczepańska, 1988; Elakovich and Wooten, 1989). *C. demersum* contains allelopathic compounds. Aqueous extracts showed inhibitory effects on seed development of *Lepidium sativum* (Kleiven and Szczepańska, 1988) and on seedling radicle growth of lettuce (Elakovich and Wooten, 1989).

All the target species are macrophytes with an allelopathic potential. To avoid a history of interactions between the target species and the water primrose, the target species *E. densa*, *M. aquaticum*, and *C. demersum* were bought in a garden center, whereas *L. hexapetala* was collected in the field. In this way, the target plants were considered “naïve” to the water primrose.

### Methods

#### Preparation of the Treatments

For this study, 100 g of small round leaves and 100 g of sediment roots of *L. hexapetala* were collected from a pond at Apigné in Brittany, France (01°44′ 25.2″ O, 48°05′ 41.4″ N), in spring at mid-March and in autumn at the end of September. The leaves and roots of *L. hexapetala* were washed to remove zooplankton and epiphytes. Aqueous extracts of live leaves and sediment roots were prepared in tap water by crushing 100 g of fresh leaves or fresh roots in 2000 mL of tap water, and the pulpy mixture was stored for 72 h at 4°C. The mixture was filtered through filter paper (Whatman #1) to remove smaller particulate matter (Elakovich and Wooten, 1989). Then it was centrifuged for 15 min at 9,000 rpm. The supernatant thus obtained constituted the aqueous extract. The leaf and root leachates of *L. hexapetala* were prepared by soaking 100 g (fresh leaves or fresh roots) in 2000 mL of tap water for 72 h in the dark at 4°C. They were then filtered through filter paper (Whatman #1). Each leachate/aqueous extract was divided into two: one part (1500 mL) was used to test the potential allelopathic effect of *L. hexapetala* on the three macrophytes species and the second (500 mL) was used to identify allelochemicals.

#### Experimental Design

The individuals of *E. densa*, *M. aquaticum*, and *C. demersum* were bought in a garden center 15 days before the beginning of the experiment in spring and in autumn (respectively, at the beginning of March and in mid-September) and acclimatized in tap water at the ambient temperature for 2 weeks. The tap water was slightly basic with a moderate nutrient concentration (mean annual value according to French Water Agency data: conductivity = 462 μS cm^-1^; pH = 7.95; [NO_3_^-^ N] = 6.95 mg L^-1^; [NH_4_^+^ N] = 0.03 mg L^-1^; [PO_4_^3-^ P] = 0.043 mg L^-1^). The amphibious *M. aquaticum* had both submerged leaves and aerial leaves which emerged above the surface of the water. After the acclimatization period, the three target plants – *C. demersum*, *M. aquaticum*, and *E. densa* – were rinsed with distilled water and their shoots cut to a length of 5 cm. All the selected shoots had an intact apex, no roots, and no trace of necrosis, buds, or lateral stems. One shoot of each plant species (*E. densa*, *C. demersum*, and *M. aquaticum*) was placed in a cylindrical plastic tube (100 mL, height: 10 cm) filled with 50 mL of the solution (i.e., leaf or root leachates, or leaf or root extracts, or tap water). The water level in each plastic tube was maintained by adding tap water, to avoid increasing allelochemical concentrations, plant desiccation and nutrient deficiencies and to offset losses from evaporation. Ten replicates were used per plant species and treatment. Plants were placed in one growth chamber (Photon Flux Density 80 μmol s^-1^ m^2^, 12 h light/12 h dark cycle, and at 16°C) for 28 days, and their position in the chamber was completely randomized. The incubation temperature of 16°C was the maximal temperature observed in spring and autumn.

#### Measured Variables

Photosynthetic yield was monitored to assess the impact of allelochemical stress responses on the photosynthesis of the target plants. To evaluate the allelopathic effect on the photosynthetic yield, a pulse amplitude modulation (PAM) chlorophyll fluorometer was used to measure photosynthetic activity. PAM is a convenient and sensitive method for monitoring photosynthetic activities. The fluorescence yield was measured on the apex leaves using a Diving-PAM underwater fluorometer (Walz) after a 30-min period of dark adaptation in the afternoon of the first day and then every week for 28 days. The initial fluorescence – Fo – and maximal fluorescence – Fm – were recorded by turning on the weak measuring light, and Fm was given after the saturation flash. The maximum quantum yield (Fv/Fm) was calculated as Fv/Fm = (Fm-Fo)/Fm with Fv variable Fluorescence.

Four morphological traits (plant stem length, roots and lateral branches length, and leaf area) of each plant were measured after 28 days of exposure to the aqueous extracts or leachates solutions in the laboratory. One picture was taken of one leaf per plant at the beginning and at the end of the experiment. The leaf area was measured using Image J software. The relative growth rate (RGR; cm d^-1^) was calculated following Hunt’s (1990) formulation: RGR = [ln(L2)-ln(L1)]/(T2-T1), in which L1 and L2 refer to stem length at time points T1 and T2. The same experimental design was applied in spring and in autumn.

### Chemical Composition of the Leaf/Root Aqueous Extracts and Leachates of *L. hexapetala*

The leaf leachates and leaf aqueous extracts of *L. hexapetala* that were not used in the allelopathy experiment, were lyophilized and ground into a powder prior to chemical analysis. The leaf leachates and aqueous extracts of *L. hexapetala* in spring and in autumn were analyzed using liquid chromatography and high resolution mass spectrometry (LCMS) according to the method described by Santonja et al. (2018). There was not enough root material after lyophilisation to conduct the analyses.

### Statistical Analysis

Photosynthetic yields were analyzed on a repeated measures basis using a non-parametric test (Naguchi et al., 2012), since the data did not meet homoscedasticity and normality requirements for parametric tests. Whenever treatment effect or the interaction between treatment and time was significant, a pairwise comparison of treatment levels and treatment levels within a given sampling time was performed using a Mann–Whitney–Wilcox test, and a Benjamini-Hochberg False Discovery Rate (10% acceptance level) correction was subsequently applied to multiple test series (Benjamini and Yekutieli, 2001). Non-parametric repeated measures analyses were performed with R software (R Core Team, 2016) and a nparLD package (Naguchi et al., 2012).

The abilities of each plant species to grow and produce roots and lateral branches under different treatments depending on the season were analyzed using a two-way linear model. The leaf area growth of *C. demersum*, as well as the length of the lateral shoots and roots of each species were log-transformed to check their residual homoscedasticity and normality. When the number of data available per trait and combination of season treatments was strictly lower than three, the combination was not included in the statistical analysis dataset. This was particularly the case for the lateral shoots and root length, as the species did not produce lateral shoots or roots in every treatment. Consequently, some treatments do not appear in the results section or in the Figures and Tables in this paper. The adequate distribution of model residuals was verified for each trait by checking the model plots. Tukey’s HSD tests were applied to observe differences between treatments. Untransformed means and standard errors were used in the Figures to facilitate interpretation. Statistical analyses were performed with R software (R Core Team, 2016).

## Results

**FIGURE 1:**
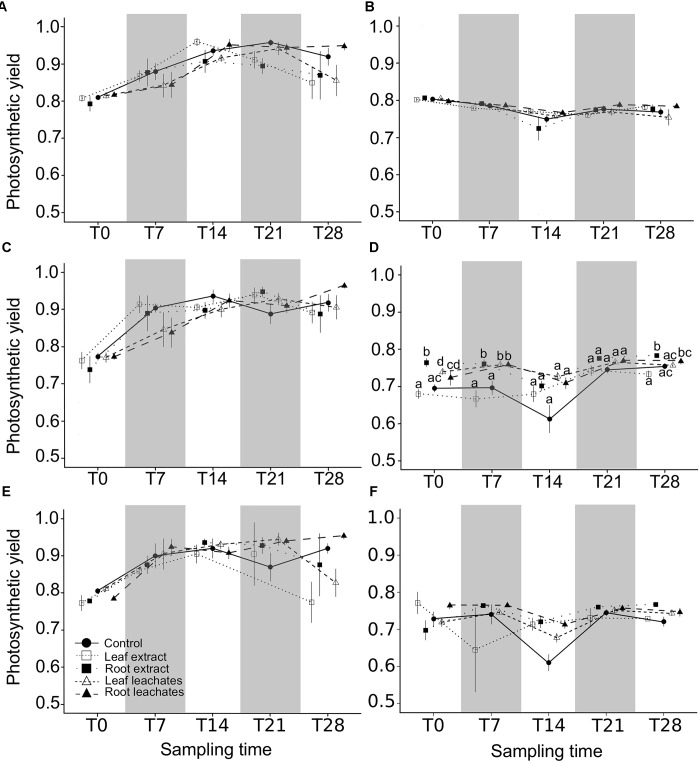
Mean photosynthetic yield plus standard error for the three species in spring and autumn: *Myriophyllum aquaticum* (**A** in spring and **B** in autumn), *Egeria densa* (**C** in spring and **D** in autumn), and *Ceratophyllum demersum* (**E** in spring and **F** in autumn). Treatments are denoted by white squares for leaf extract treatments, black squares for root extract treatments, white triangles for leaf leachate treatments, black triangles for root leachate treatments, and black circles for control series. Symbols marked with the same letter are not significantly different for treatment factors within the same period (*p* > 0.05), according to Mann–Whitney–Wilcoxon pairwise comparisons significance test adjusted by the Benjamini-Yekutieli correction.

**FIGURE 2:**
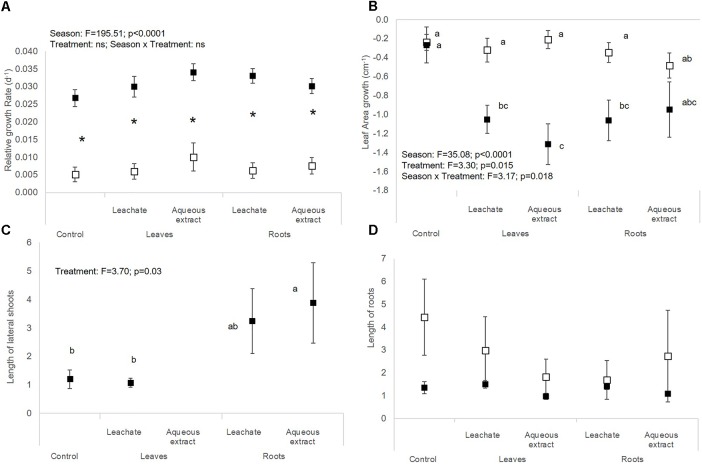
**(A)** Relative growth rate, **(B)** leaf area growth, **(C)** length of lateral shoots, and **(D)** length of roots of *M. aquaticum* (mean ± SE) in spring (white symbols) and autumn (black symbols) depending on the treatment (i.e., control, leaf leachates, leaf aqueous extracts, root leachates, root aqueous extracts) after a 28-day experiment in the laboratory. Different small letters indicate significant differences between the interaction season × treatment. Stars show significant differences between seasons.

**FIGURE 3:**
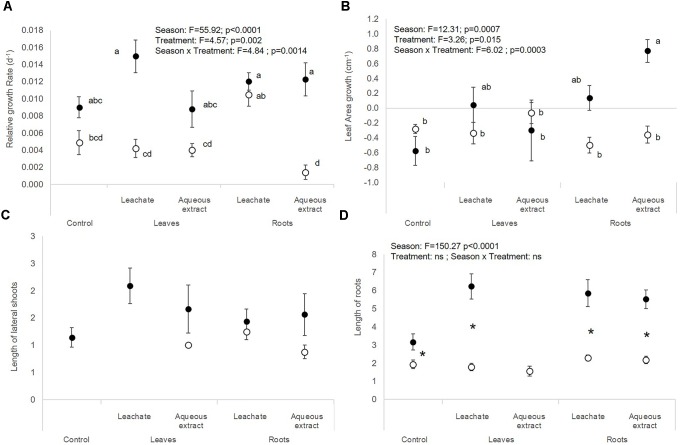
**(A)** Relative growth rate, **(B)** leaf area growth, **(C)** length of lateral shoots, and **(D)** length of roots of *E. densa* (mean ± SE) in spring (white symbols) and autumn (black symbols) depending on the treatment (i.e., control, leaf leachates, leaf aqueous extracts, root leachates, roots aqueous extracts) after a 28-day experiment in the laboratory. Different small letters indicate significant differences between the interaction season × treatment. Stars show significant differences between seasons.

**FIGURE 4:**
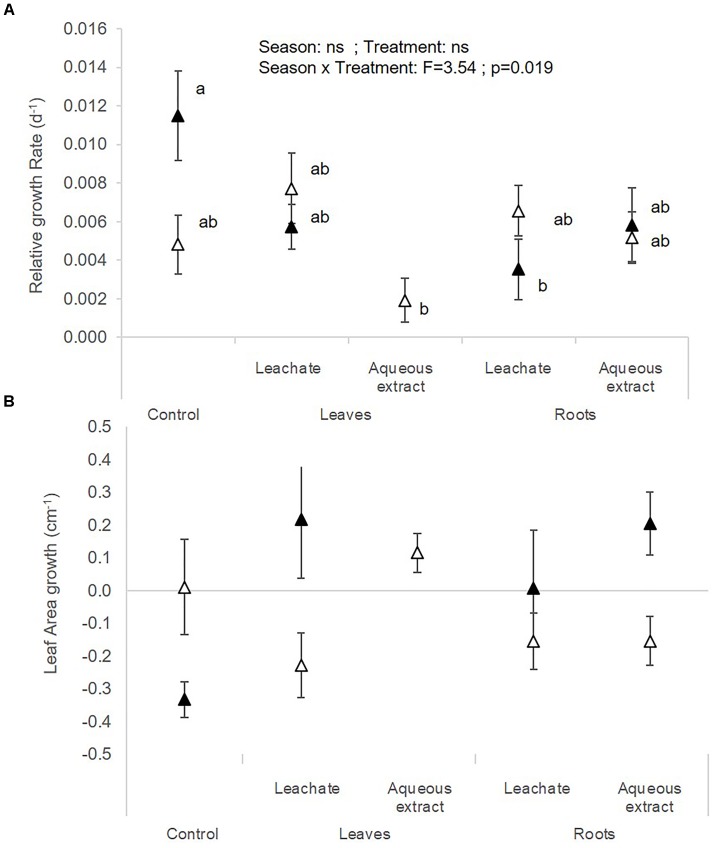
**(A)** Relative growth rate and **(B)** leaf area growth of *C. demersum* (mean ± SE) in spring (white symbols) and autumn (black symbols) depending on the treatment (i.e. control, leaf leachates, leaf aqueous extracts, root leachates, root aqueous extracts) after a 28-day experiment in the laboratory. Different small letters indicate significant differences between the interaction season × treatment.

There were no significant effects of spring aqueous extracts and leachates on the photosynthetic yield or on the morphological traits of *M. aquaticum*, *E. densa*, and *C. demersum* (Tables 1, 2 and Figures 1–4). Moreover, aqueous extracts and leachates had no significant effect on the photosynthetic yield of *M. aquaticum* in autumn (Table 1 and Figures 1A,B). The RGR of *M. aquaticum* was higher in autumn than in spring (*F* = 195.51; *p* < 0.0001, Figure 2A), although it was not impacted by the treatments (Table 2). Leaf area growth was affected negatively in autumn by the leaf treatments and by the root leachates (*F* = 3.17; *p* = 0.018, Figure 2B). The lengths of lateral shoots of *M. aquaticum* were longer after exposure to the root extracts (*F* = 3.89; *p* = 0.027, Figure 2C) than after exposure to leaf treatments.

**Table 1:** Effects of leachates and extracts of *Ludwigia hexapetala* on the photosynthetic yield of target species in spring and in autumn.

		Autumn			Spring		
		ATS	df	*p*	ATS	df	*p*
*Myriophyllum aquaticum*	Treatment	0.73	3.23	0.6	0.96	2.37	0.4
	Time	39.67	3.02	**<0.0001**	39.18	3.30	**<0.0001**
	Treatment × time	1.14	8.75	0.3	1.65	8.03	0.1
*Egeria densa*	Treatment	16.11	3.85	**<0.0001**	0.19	3.88	0.9
	Time	40.56	3.58	**<0.0001**	25.21	2.86	**<0.0001**
	Treatment × time	2.68	10.66	**0.002**	1.24	7.81	0.3
*Ceratophyllum demersum*	Treatment	5.09	2.66	**0.003**	1.94	3.31	0.1
	Time	6.67	1.69	**0.002**	11.93	2.43	**<0.0001**
	Treatment × time	1.20	2.01	0.3	1.4	3.5	0.2

*The significant differences are indicated in bold type; ATS denotes ANOVA Type Statistic.*

**Table 2:** Summary of the two-factor linear model, with season and treatment as factors, on the different morphological traits measured for each species (*C. demersum*, *E. densa*, and *M. aquaticum*) in the laboratory experiment (with the degrees of freedom (Df), the range of the number of replicates per modalities for each factor (n), *F*-values for each factor per trait, and the significance level (p).

		Relative growth rate			Leaf area growth			Length of lateral shoots			Length of roots		
	Df	*n*	*F*	*p*	*n*	*F*	*p*	*n*	*F*	*p*	*n*	*F*	*p*
***C. demersum***													
Season	1	31–50	0.55	0.46	29–50	2.00	0.16	/	/	/	/	/	/
Treatment	4	10–20	2.30	*0.07*	10-20	1.19	0.32	/	/	/	/	/	/
Season × treatment	3	6–10	3.54	**0.02**	4–10	2.72	*0.051*	/	/	/	/	/	/
***E. densa***													
Season	1	46–50	55.92	**<0.0001**	44–50	12.31	**<0.001**	11–34	3.03	*0.09*	31–35	150.27	**<0.0001**
Treatment	4	16–20	4.57	**0.002**	16–20	3.26	**0.015**	6–12	1.35	0.27	6–19	2.51	*0.052*
Season × treatment	4	6–10	4.84	**0.001**	6–10	6.02	**<0.001**	3–8	0.48	0.62	3–10	1.98	0.13
***M. aquaticum***													
Season	1	42–48	195.51	**<0.0001**	40–49	35.08	**<0.0001**	/	/	/	23–27	1.43	0.24
Treatment^∗^	4	15–20	1.39	0.25	16–20	3.30	**0.015**	4–7	3.70	**0.03**	7–14	0.87	0.49
Season × treatment	4	6–10	0.26	0.90	6–10	3.17	**0.02**	/	/	/	3–9	0.62	0.65

*Significant results are in bold type; tendencies are in italic. ^∗^Df = 3 for the length of lateral shoots.*

The autumnal root treatments and the leaf leachates stimulated the photosynthetic yield of *E. densa* after 7 and 28 days in the plants exposed to the root extracts (interaction treatment × sampling date, Table 1 and Figures 3C,D). RGR and leaf area growth of *E. densa* were affected by the interactions between the treatment and the season (Table 2), but the length of the roots only depended on the season (Table 2). The autumnal root extract stimulated the growth of the *E. densa* leaves (interaction season × treatment: *F* = 6.02; *p* = 0.0003, Figure 3B). The lengths of the roots (*F* = 150.27; *p* < 0.0001, Figure 3D) were higher in autumn than in spring (Table 2).

The photosynthetic yield of *C. demersum* was stimulated by the root extracts in autumn (Table 1 and Figure 1F). Relative growth rate (RGR) of *C. demersum* was particularly affected by the interaction season × treatment (*F* = 3.54; *p* = 0.019, Table 2). Indeed, the root leachates of *L. hexapetala* decreased the RGR of *C. demersum* in autumn (Figure 4A). In contrast, the growth of the leaf area tended to be higher when *C. demersum* was exposed to the root treatments than the control treatment in autumn (interaction season × treatment: *F* = 2.72; *p* = 0.051, Figure 4B). The Coontail (*C. demersum*) produced no roots and very few lateral branches both in spring and autumn (Table 2).

Three main allelochemicals were identified in the leaf leachates and extracts of *L. hexapetala*: quercitrin, prunin, myricitrin. Concentrations of these allelochemicals were greater in the leaf extracts than in the leaf leachates (Table 3). They were higher in autumn than in spring (Table 3).

**Table 3:** Mean concentrations (± SE; *n* = 3; μg L^-1^) of chemical compounds found in the leaf aqueous extracts and leachates of *Ludwigia hexapetala* in spring and in autumn.

	Spring	Autumn
**Leaf leachates**		
Myricitrin	88.0 +/- 9.1^a^	334.4 +/- 9.2^b^
Prunin	56.6 +/- 0.6^a^	121.5 +/- 3.4^b^
Quercitrin	97.7 +/- 1.7^a^	141.9 +/- 4.1^b^
**Leaf aqueous extract**		
Myricitrin	2538^a^	8899^b^
Prunin	2185^a^	3187^b^
Quercitrin	2877^a^	3981^b^

*One-way ANOVAs were performed for differences between seasons for each chemical compound. F-values and associated P-values are indicated. Different letters denote significant differences between the two seasons with a < b (post hoc Tukey tests results after one-way ANOVA); d.f., degree of freedom.*

## Discussion

### Potential Allelopathic Effects of *L. hexapetala* on the Growth of Other Macrophyte Species

This study aimed to assess whether the aqueous leaf/root extracts or the leaf/root leachates of the *L. hexapetala* had a positive or negative effect on the development of other macrophyte species. Our results showed that the root extracts stimulated the lateral branches of *M. aquaticum* and the leaf area of the two submerged species *C. demersum* and *E. densa*. Conversely, the leaf treatments and root leachates inhibited the leaf area growth of *M. aquaticum*. The latter result could be explained by similar biological type and niche overlap: *L. hexapetala* and *M. aquaticum* are both exotic species with similar growth forms (amphibious) native from the same geographical range. Although the two species co-exist in the field, their patches are spatially separated. In a previous study, we found that *L. hexapetala* stimulated the root production and the growth of *M. aquaticum* at low densities (Thouvenot et al., 2013b). The allelochemicals released by roots of *L. hexapetala* could directly favor the lateral growth of *M. aquaticum* (length of lateral shoots) or could also indirectly affect its development by modifying the chemical and physical properties of the soil/sediment and by regulating the soil microbial community (Walker et al., 2003). Parrot’s Feather (*M. aquaticum*) may have the capacity to protect itself from allelopathy by metabolizing certain phenolic compounds with allelopathic activity (Elakovich and Wooten, 1989). This ability of Parrot’s Feather to synthesize phenols gives it an advantage in allelopathic interactions and may favor its competitiveness. Further studies testing the effect of *M. aquaticum* root/leaf leachates on *L. hexapetala* are required.

However, we observed that the effects of the root and leaf treatments differ according to the biological growth forms of the exotic species. The biological type of plants affects levels of secondary compounds. For example, emergent species in wetlands contain more phenolics than submerged plant species (Smolders et al., 2000). In our experiment, there was no effect of treatments on the apical growth (RGR) of *E. densa*, a species from the same geographical area as the water primrose. Previously, we had established that there was no competition between *E. densa* and *L. hexapetala*, but that there was facilitation (Thouvenot et al., 2013b). The secondary compounds produced by the roots of the water primrose stimulated leaf area growth and consequently had a positive impact on the photosynthetic yield. Our results suggested positive interactions between the water primrose and the submerged *E. densa*. In contrast, the growth of the native submerged plant *C. demersum* was inhibited by root leachates. This result is congruent with the “Novel Weapons Hypothesis” (Callaway and Ridenour, 2004) and with the literature (Sakpere et al., 2010). Previously, Kulshretha and Gopal (1983) established that *C. demersum* and *C. muricatum* were negatively affected by the exotic submerged *Hydrilla verticillata*. Moreover, Sakpere et al. (2010) observed that exudates of *Ludwigia decurrens* and *Ludwigia adscendens* reduced the stem length of *Corchorus olitorius* seedlings in early growth.

### Seasonal Effect of *L. hexapetala* on the Other Macrophyte Species

The autumnal treatments had significant effects on the photosynthetic yield and on the morphological traits of the two submerged species and the exotic amphiphyte, whereas no spring effects were established. The variation in significance of the effects of *L. hexapetala*, depending on the time at which the roots and the leaves were collected, confirmed the hypothesis that the plants change seasonally. Previous studies have also reported a seasonal pattern of allelopathic interactions (Bauer et al., 2009; Silva et al., 2014). For example, Bauer et al. (2009) showed an optimal inhibitory effect of *Myriophyllum verticillatum* on the cyanobacteria *Anabaena variabilis* in spring. Indeed, the secondary compound composition in *L. hexapetala* could be different in spring and autumn (Dandelot et al., 2008; Santonja et al., 2018) due to variations in the environmental conditions of the Apigné ponds (i.e., nutrients, light intensity or water depth), of climatic parameters (Chaves and Escudero, 1999) and of the plant phenological stage. The presence of allelochemicals produced by short leaves is dependent on the season (Dandelot et al., 2008). The seasonal fluctuations could alter the allelopathic activity of the secondary compounds (Santonja et al., 2018). Our results should be used with caution because the different impact of aqueous extracts and leachates according to the season could be explained both by seasonal fluctuations in the physiology of the target species (*C. demersum*, *E. densa*, *M. aquaticum*) and of the donor species *L. hexapetala.* However, our target species are clonal individuals, cultivated under glasshouse, conditions which should reduce the fluctuations due to plant phenology. Furthermore, the absence of a seasonal fluctuation in the RGR of *E. densa* and of *C. demersum* in the control suggested that the seasonal fluctuation of donor species *L. hexapetala* is the basis of potential seasonal effects of the allelopathy. The growth rate of *M. aquaticum* was not affected by allelopathy but only by season. The ability of *M. aquaticum* to metabolize phenolic compounds could counteract the impact of high concentrations of allelochemicals produced by the water primrose in autumn.

### Chemical Composition of the Leaf/Root Aqueous Extracts and Leachates of *L. hexapetala*

Three main flavonoids belonging to the polyphenol family were identified in the leaf treatments in spring and in autumn: quercitrin, prunin, myricitrin. The allelochemical composition is phylogenetically determined (Grutters et al., 2017). Numerous compounds are produced by *Ludwigia* sp.: saponins, tannins, polyphenols, alkaloids, linoleic acids, and flavonoids (Averett et al., 1990). However, phenolics are the compounds most frequently involved in allelopathy in freshwaters (Gross, 2003; Iason et al., 2013) or between aquatic plant species (Dandelot et al., 2008). According to an analysis of root extract conducted by Marcellin-Gros (2015), the most abundant secondary compounds in *L. hexapetala* are two tannins (pedunculagin and an ellagic acid) and the flavonoid quercetin. The result found by Marcellin-Gros (2015) suggested that composition of the root extracts differed from that of the leaf treatments, except for quercitrin. Quercitrin had a positive effect on the photosynthetic yield of phytoplankton (Santonja et al., 2018), and was higher in the root extracts of *L. hexapetala* (Marcellin-Gros, 2015) than in leaf extracts. This secondary compound may have been released into the water by the roots of the water primrose and could have stimulated the photosynthetic yield and the leaf area of the two submerged species *E. densa* and *C. demersum*. Previous work has suggested that the tannin pedunculagin is characterized by its antioxidant properties and its positive effects on human health (Biswas et al., 2014), whereas ellagic acid is a rooting inhibitor (Viéitez and Ballester, 1986; Qin et al., 2006). However, there was no root inhibition detected for *E. densa* and *M. aquaticum*. Thus, the roles of these two tannins are unknown and need to be investigated further.

The concentrations of the allelochemicals in the leaves were higher in autumn, which may be related to the seasonal fluctuations of environmental parameters and trade-offs between primary and secondary metabolisms. In autumn, plants are exposed to a decrease in temperatures and solar radiation, which has been reported to have an inverse relationship with secondary metabolite accumulation in plant tissues (Silva et al., 2014).

## Conclusion

The first hypothesis of this study was partially validated for *M. aquaticum* and for *C. demersum*, indicating an autumnal phytotoxic effect of leaf treatments on the leaf area growth of the exotic Parrot’s Feather and of root leachates on the growth of the native species. However, *L. hexapetala* favors the growth of lateral shoots of *M. aquaticum* and the leaf area and photosynthetic yield of *E. densa*; suggesting a facilitation effect of the root treatment on the two other exotic species. In contrast, *L. hexapetala* strongly reduced the growth of the native plant. However, many plants release allelochemicals into the environment with little impact on the performance of native plants, due to a long coevolutionary history (Thorpe et al., 2009). Indeed, there are several biotic and abiotic factors (interactions between plants and herbivores/pathogens, climatic conditions) that are able to change allelopathic impact on the recipient community (Inderjit et al., 2011).

Our second hypothesis, that the impact of leaf and root treatments on the growth of the three target species showed seasonal fluctuations, was validated. There was no impact of the treatment in spring. Plant growth is optimal in spring. There are trade-offs between the primary biological functions of plants, such as growth, and resource allocation for chemical defense (Herms and Mattson, 1992). The variation in the effects of *L. hexapetala* demonstrated in this study highlights the importance of conducting allelopathy research during different seasons; if this variation is not considered, the results may not reflect the potential effect of a plant species correctly (Silva et al., 2014). Moreover, the seasonal dependence of biotic interactions has not been studied in depth and it is necessary to take this into account in order to gain a better understanding of the interactions between native and exotic macrophyte species and between different exotic species.

## Author Contributions

GT and LT designed and conducted the experiments. LT and HR-P analyzed the data. GT wrote the manuscript with contributions from LT and HR-P.

## Conflict of Interest Statement

The authors declare that the research was conducted in the absence of any commercial or financial relationships that could be construed as a potential conflict of interest.

## References

[B1] Agami M. Waisel Y. (1985). Inter-relationship between *Najas marina* L. and three other species of aquatic macrophytes. *Hydrobiologia* 126 169–173. 10.1007/BF00008684

[B2] Averett J. E. Zardini E. M. Hoch P. C. (1990). Flavonoid systematics of ten sections of *Ludwigia* (Onagraceae). *Biochem. Syst. Ecol.* 18 529–532. 10.1016/0305-1978(90)90124-X

[B3] Bauer N. Blaschke U. Beutler E. Gross E. M. Jenett-Siems K. Siems K. (2009). Seasonal and interannual dynamics of polyphenols in *Myriophyllum verticillatum* and their allelopathic activity on *Anabaena variabilis*. *Aquat. Bot.* 91 110–116. 10.1016/j.aquabot.2009.03.005

[B4] Benjamini Y. Yekutieli D. (2001). The control of the false discovery rate in multiple testing under dependency. *Ann. Stat.* 29 1165–1188. 18298808

[B5] Biswas T. K. Chakrabarti S. Pandit S. Dey S. K. (2014). Pilot study evaluating the use of *Emblica officinalis* standardized fruit extract in cardio-respiratory improvement and antioxidant status of volunteers with smoking history. *J. Herb. Med.* 4 188–194. 10.1016/j.hermed.2014.09.002

[B6] Callaway R. M. Ridenour W. M. (2004). Novel weapons: invasive success and the evolution of increased competitive ability. *Front. Ecol. Environ.* 2 436–443.

[B7] Chaves N. Escudero J. C. (1999). “Variation of flavonoid synthesis induced by ecological factors,” in *Principles and Practices in Plant Ecology: Allelochemical Interactions*, eds Inderjit, K. M. N. Dakshini, and F. L. Chester (Boca Raton, FL: CRC Press), 267–285.

[B8] Cook C. D. K. Urmi-König K. (1984). A revision of the Genus *Egeria* (Hydrocharitaceae). *Aquat. Bot.* 19 73–96. 10.1016/0304-3770(84)90009-3

[B9] Dandelot S. Robles C. Pech N. Cazaubon A. Verlaque R. (2008). Allelopathic potential of two invasive alien *Ludwigia* spp. *Aquat. Bot.* 88 311–316. 10.1016/j.aquabot.2007.12.004

[B10] Denny P. (1972). Sites of nutrient absorption in aquatic macrophytes. *J. Ecol.* 60 819–829. 10.2307/2258568 29299657

[B11] Elakovich S. D. Wooten J. W. (1989). Allelopathic potential of sixteen aquatic and wetland plants. *Toxicology* 17 129–182.

[B12] Espinosa-Rodríguez C. A. Rivera-De la Parra L. Martínez-Téllez A. Gomez-Cabral G. C. Sarma S. S. S. Nandini S. (2016). Allelopathic interactions between the macrophyte *Egeria densa* and plankton (alga, *Scenedesmus acutus* and cladocerans, *Simocephalus* spp.): a laboratory study. *J. Limnol.* 75 151–160. 10.4081/jlimnol.2016.1397

[B13] Fernandez O. A. Sutton D. Lallan V. H. Sabbatini M. R. (1993). “Aquatic weed problems and management in South and Central America,” in *Aquatic Weeds. Ecology and Management of Nuisance of Aquatic Vegetation*, eds Pieterse A. Murphy K. (Oxford: Oxford University Press), 406–425.

[B14] Gatti A. B. Takao L. K. Pereira V. C. Ferreira A. G. Lima M. I. S. Gualtieri S. C. J. (2014). Seasonality effect on the allelopathy of *Cerrado* species. *Braz. J. Biol.* 74 (Suppl. 3), 64S–69S. 10.1590/1519-6984.21512 25627367

[B15] Gopal B. Goel U. (1993). Competition and allelopathy in aquatic plant communities. *Bot. Rev.* 59 155–210. 10.1007/BF02856599 17918398

[B16] Gross E. M. (2003). Allelopathy of aquatic autotrophs. *Crit. Rev. Plant Sci.* 22 313–339. 10.1080/713610859 11351765

[B17] Grutters B. Saccomanno B. Gross E. M. Van de Waal D. B. van Donk E. Bakker E. S. (2017). Growth strategy, phylogeny and stoichiometry determine the allelopathic potential of native and non-native plants. *Oikos* 126 1770–1779. 10.1111/oik.03956

[B18] Helmig D. Daly R. W. Milford J. Guenther A. (2013). Seasonal trends of biogenic terpene emissions. *Chemosphere* 93 35–46. 10.1016/j.chemosphere.2013.04.058 23827483

[B19] Herms D. Mattson W. (1992). The dilemma of plants - to grow or defend. *Q. Rev. Biol.* 67 283–335. 10.1086/417659

[B20] Hunt R. (1990). *Basic Growth Analysis.* London: Unwin Hyman 10.1007/978-94-010-9117-6

[B21] Hussner A. (2009). Growth and photosynthesis of four invasive aquatic plant species in Europe. *Weed Res.* 49 506–515. 10.1111/j.1365-3180.2009.00721.x

[B22] Iason G. R. Dicke M. Hartley S. E. (2013). *The Ecology of Plant Secondary Metabolites: From Genes to Global Processes.* New York, NY: Cambridge University Press.

[B23] Inderjit Wardle D. A. Karban R. Callaway R. M. (2011). The ecosystem and evolutionary contexts of allelopathy. *Trends Ecol. Evol.* 26 655–662. 10.1016/j.tree.2011.08.003 21920626

[B24] Kim Y. O. Lee E. J. (2011). Comparison of phenolic compounds and the effects of invasive and native species in East Asia: support for the novel weapons hypothesis. *Ecol. Res.* 26 87–94. 10.1007/s11284-010-0762-7

[B25] Kleiven S. Szczepańska W. (1988). The effects of extracts from *Chara tomentosa* and two other aquatic macrophytes on seed germination. *Aquat. Bot.* 32 193–198. 10.1016/0304-3770(88)90099-X

[B26] Kulshretha M. Gopal B. (1983). Allelopathic influence of *Hydrilla verticillata* (L.F.) royle on the distribution of *Ceratophyllum* species. *Aquat. Bot.* 16 207–209. 10.1016/0304-3770(83)90095-5

[B27] Les D. H. Merhoff L. J. (1999). Introduction of nonindigenous aquatic vascular plants in southern New England: a historical perspective. *Biol. Invasions* 1 281–300. 10.1023/A:1010086232220

[B28] Lombardo P. Mjelde M. Källqvist T. Brettum P. (2013). Seasonal and scale-dependent variability in nutrient- and allelopathy-mediated macrophyte–phytoplankton interactions. *Knowl. Manag. Aquat. Ecosyst.* 409:31 10.1051/kmae/2013055

[B29] Marcellin-Gros R. (2015). *Caractérisation des Métabolites Secondaires des Différentes Formes de Croissances Chez Ludwigia Grandiflora.* Master Report. Lyon: Université Claude Bernard, 25.

[B30] Naguchi K. Gel Y. R. Brunner E. Konietschke F. (2012). nparLD: an R software for the nonparametric analysis of longitudinal data in factorial experiments. *J. Stat. Softw.* 50 1–23. 10.18637/jss.v050.i1225317082

[B31] Nakai S. Inoue Y. Hosomi M. Murakami A. (1999). Growth inhibition of blue-green algae by allelopathic effects of macrophytes. Water Sci. Technol. 39 47–53. 10.1016/S0273-1223(99)00185-7

[B32] Petrussa E. Braidot E. Zancani M. Peresson C. Bertolini A. Patui S. (2013). Plant flavonoids—biosynthesis, transport and involvement in stress responses. *Int. J. Mol. Sci.* 14 14950–14973. 10.3390/ijms140714950 23867610 PMC3742282

[B33] Qin B. Perry L. G. Broeckling C. D. Jiang D. J. Stermitz F. R. Paschke M. W. (2006). Phytotoxic allelochemicals from roots and root exudates of leafy spurge (*Euphorbia esula*). *Plant Signal. Behav.* 1 323–327. 10.4161/psb.1.6.3563 19517003 PMC2634247

[B34] R Core Team (2016). *R: A Language and Environment for Statistical Computing.* Vienna: R Foundation for Statistical Computing.

[B35] Rice E. L. (1984). *Allelopathy*, 2nd Edn. New York, NY: Academic Press.

[B36] Sakpere A. M. Oziegbe M. Bilesanmi I. A. (2010). Allelopathiceffectsof *Ludwigia decurrens* and L.*adscendens* subsp. *diffusa* on germination, seedling growth and yield of *Corchorus olitorius* L. *Not. Sci. Biol.* 2 75–80. 10.15835/nsb224629

[B37] Santonja M. Le Rouzic B. Thiébaut G. (2018). Seasonal dependence and functional implications of macrophyte-phytoplankton allelopathic interactions. *Freshw. Biol.* 63 1161–1172. 10.1111/fwb.13124

[B38] Sheppard A. W. Shaw R. H. Sforza R. (2006). Top 20 environmental weeds for classical biological control in Europe review of opportunities adoption regulations and other barriers to. *Weed Res.* 46 93–117. 10.1111/j.1365-3180.2006.00497.x

[B39] Silva E. R. Overbeck G. E. Soares G. L. G. (2014). Phytotoxicity of volatiles from fresh and dry leaves of two Asteraceae shrubs: evaluation of seasonal effects. *S. Afr. J. Bot.* 93 14–18. 10.1016/j.sajb.2014.03.006

[B40] Simberloff D. Von Holle B. (1999). Positive interactions of nonindigenous species: invasional meltdown? *Biol. Invasions* 1 21–32. 10.1023/A:1010086329619

[B41] Smolders A. J. P. Vergeer L. H. T. Van der Velde G. Roelofs J. G. M. (2000). Phenolic contents of submerged, emergent and floating leaves of aquatic and semi-aquatic macrophyte species: why do they differ? *Oikos* 91 307–310. 10.1034/j.1600-0706.2000.910211.x

[B42] Stiers I. Crohain N. Josens G. Triest L. (2011). Impact of three aquatic invasive species on native plants and macroinvertebrates in temperate ponds. *Biol. Invasions* 13 2715–2726. 10.1007/s10530-011-9942-9

[B43] Thorpe A. S. Thele G. C. Diaconu A. Callaway R. M. (2009). Root exudate is allelopathic in invaded community but not in native community: field evidence for the novel weapons hypothesis. *J. Ecol.* 97 641–645. 10.1007/s10886-011-0005-6 21882071

[B44] Thouvenot L. Haury J. Thiebaut G. (2013a). A success story: water primroses, aquatic plant pests. *Aquat. Conserv. Mar. Freshw. Ecosyst.* 23 790–803. 10.1002/aqc.2387

[B45] Thouvenot L. Puech C. Martinez L. Haury J. Thiébaut G. (2013b). Strategies of the invasive macrophyte *Ludwigia grandiflora* in its introduced range: competition, facilitation or coexistence with native and exotic species? *Aquat. Bot.* 107 8–13. 10.1016/j.aquabot.2013.01.003

[B46] Vanderstukken M. Mazzeo N. van Colen W. Declerck S. A. J. Muylaert K. (2011). Biological control of phytoplankton by the subtropical submerged macrophytes *Egeria densa* and *Potamogeton illinoensis*: a mesocosm study. *Freshw. Biol.* 56 1837–1849. 10.1111/j.1365-2427.2011.02624.x

[B47] Viéitez F. J. Ballester A. (1986). Presence of root inhibitors in chestnut cuttings. *Bol. Acad Galega de Ciencias* 5 125–132.

[B48] Walker T. S. Bais H. P. Grotewold E. Vivanco J. M. (2003). Root exudation and rhizosphere biology. *Plant Physiol.* 132 44–51. 10.1104/pp.102.019661 12746510 PMC1540314

